# Effect of Furin inhibitor on lung adenocarcinoma cell growth and metastasis

**DOI:** 10.1186/1475-2867-14-43

**Published:** 2014-05-22

**Authors:** Yong-Chao Ma, Wen-Juan Fan, Shu-Mei Rao, Li Gao, Zhan-Yu Bei, Song-Tao Xu

**Affiliations:** 1 Luo He Medical Colledge, Daxue Road, #148, Luohe City, Henan Province 462002, P.R. China

**Keywords:** Fruin inhibitors, Lung adenocarcinoma, Apoptosis, Migration

## Abstract

**Background:**

To investigate the mechanisms of lung adenocarcinoma cell metastasis and provide a theoretical basis for the in-depth study of lung adenocarcinoma.

**Methods:**

A549 cells are incubated with different concentrations of Furin inhibitor for indicated times. The proliferation and migration were confirmed with MTT, colony formation, wound Healing and Transwell assayes. Hochest 33342 / PI double staining was used to detect apoptosis. Cell migration and apoptosis associated proteins were analysed by enzyme-linked immunosorbent assay (ELISA) and western blot.

**Results:**

We have found that Furin inhibitor play a significant role in inhibition A549 cell growth. And we also found cell migration was inhibited significantly upon Furin inhibitor treatment.

**Conclusion:**

The proliferration and migration of A549 cell were inhibited by Furin inbitor through down-regulation the expression of migration and apoptosis related proteins.

## Background

Lung cancer is one of the most serious threats to human health in the world, and the incidence is increasing year by year [1]. It was estimated that the number of the patients dying from lung cancer was larger than the total number of patients dying from prostate cancer and colorectal cancer one year [2]. Metastasis and recurrence is the leading cause of death in patients with lung cancer, about 90% of the lung cancer patients died from tumor metastasis [3]. Thus depth study on the molecular mechanism of lung cancer invasion and metastasis is necessary.

Furin is an important member of the family of pro-protein processing enzyme and highly expressed in a variety of tumors [4-6]. Many proteins which are closely related to tumor development, including Notch, Wnt, MT1-MMP, VEGF, etc., must be cut by Furin [4,7]. So, Furin expression can be used as the marker of tumor progression or as prognostic indicators [8,9]. Mbikay, etc. [10] studies have shown that Furin is highly expressed in lung cancer, and is closely related to lung cancer development. In our present study, we apply Furin inhibitor a1-PDX to detect the effect on the migration and invasion of A549 cells.

## Materials and methods

Human lung adenocarcinoma cell line A549 was purchased from Chinese Academy of Medical Sciences Peking Union cell libraries, cultured in RPMI1640 medium (Gibco, USA) contained with 10% fetal bovine (FBS), 100 units/mL penicillin and 100 μg/mL streptomycin at 37°C, 5% CO_2_ and humidity.

Furin inhibitor a1-PDX (Merck No.126850-2.5 MG) was dissolved in DMSO. Rabbit anti-human VEGF-C, VEGF-D, MT1-MMP, Caspase-3, Caspase-9, Bcl-2 and GAPDH antibodies were purchased from Santa Cruz Biotechnology (Santa Cruz, USA). Anti-rabbit IgG-HRP were purchased from Sigma (Sigma, USA); MTT, Hochest 33342, Transwell kit were purchased from Promega Corporation; MMP2, MMP9 ELISA kits were purchased from Nanjing Jiancheng Biological Reagent Company. Other reagents were of analytical grade.

### Cell proliferation studies by MTT assay

A549 cells in logarithmic growth phase were seeded in 96-well plate (5 × 10^3^ per well) for 24 h. Various concentrations of a1-PDX (200 nM, 400 nM) were added, then cultured for 24 h~96 h. MTT reagent (5 mg/mL) was added to each well and incubated for 4 h at 37°C. The formazan crystals were solubilized by the addition of 150 μL DMSO to each well. The optical density at 490 nm was measured and cell viability was determined by the growth curve.

### Colony formation ability of A549 cell

Monolayer cultured cells were pipetted into individual cells, then suspended in 10% FBS-containing RPMI 1640 medium, after that we inoculated these cells in petri dishes. Different concentrations of a1-PDX (200 nM, 400 nM) were added and incubated for 2 weeks. Then immobilized and stained for 10 min by Giemsa dye. After washing and air drying, clone numbers were directly counted and analyzed.

### Hochest 33342 / PI staining for apoptosis analysis

A549 cells were treated by different concentrations of a1-PDX for 48 h and washed twice with cold PBS, then incubated with 5 μL DAPI /PI staining buffer for 15 min at 25°C in the dark. The percentage of cells undergoing apoptosis was determined.

### Monolayer cell wound healing assay

The 100% confluent monolayer cells were scraped with sterile 200 μL pipette tip and cell debris was washed with PBS. The cells migrated into the wounded areas and these process were photographed at the indicated times with an inverted microscopy equipped with a digital camera. The extent of healing was defined as the ratio of the difference between the original and the remaining wound areas compared with the original wound area.

### Transwell invasion assay

A549 cells (1 × 10^5^) were incubated with different concentrations of a1-PDX on the upper chamber of the transwell, containing 200 μL of RPMI1640 medium without 10% FBS. Transwell lower chamber is filled with 500 μL of complete RPMI1640 medium containing 10% FBS. The cells were allowed to migrate for 48 h, then fixed and stained with 0.1% crystal violet for 10 min. The migrated clones were photographed under an optical microscope. The cell numbers were counted at 12 different areas.

### Western blot analysis

A549 cells were treated and collected as described above. Cells were lysed in RIPA buffer (50 mM Tris (pH 7.4), 150 mM NaCl, 1% Triton X-100, 0.1% SDS, 1% sodium deoxycholate, 5 mM EDTA, 100 mM NaF, and 1 mM Na_3_VO_4_) containing protease inhibitor cocktail for 30 min on ice, and then centrifuged for 30 min at 30000 g. Equal total proteins were electrophoresis by 12% SDS-PAGE gel, followed by transferred to PVDF membranes using a wet transblot system (Bio-Rad, Hercules, CA). The membranes were blocked for 1 h at room temperature with 5% nonfat dry milk and incubated overnight at 4°C with antibodies against MT1-MMP, VEGF-C, VEGF-D and GAPDH (1:1000). After washing, membrane was incubated for 1 h with HRP-conjugated goat anti-rabbit secondary antibody diluted 1:5,000 in PBST. After further washing and processing using Super Signal West Pico chemiluminescent substrate (Pierce, USA), the membrane was exposed to Fujifilm LAS3000 Imager (Fuji, Japan). The band densities of the western blots were normalized and compared to the relevant GAPDH band density with Image J Analyst software (NIH).

### Enzyme-linked immunosorbent assay

A549 cells were treated as previously described. Concentrations of MMP-9, MMP-2, VEGF in the cell culture supernatants were quantified using ELISA kits (R&D Systems, USA). Each sample was repeated 5 times.

### Statistical analysis

The experiments were carried out in triplicate and the data were compared to control group. Statistical analysis was performed by SPSS13.0, measurement data using paired t-test and ANOVA. A *P* value of less than 0.05 was thought to indicate statistical significance.

## Results

### *Effects of* a1-PDX *on proliferation and colony formation abilities of* A549 *cell*

**Figure 1:**
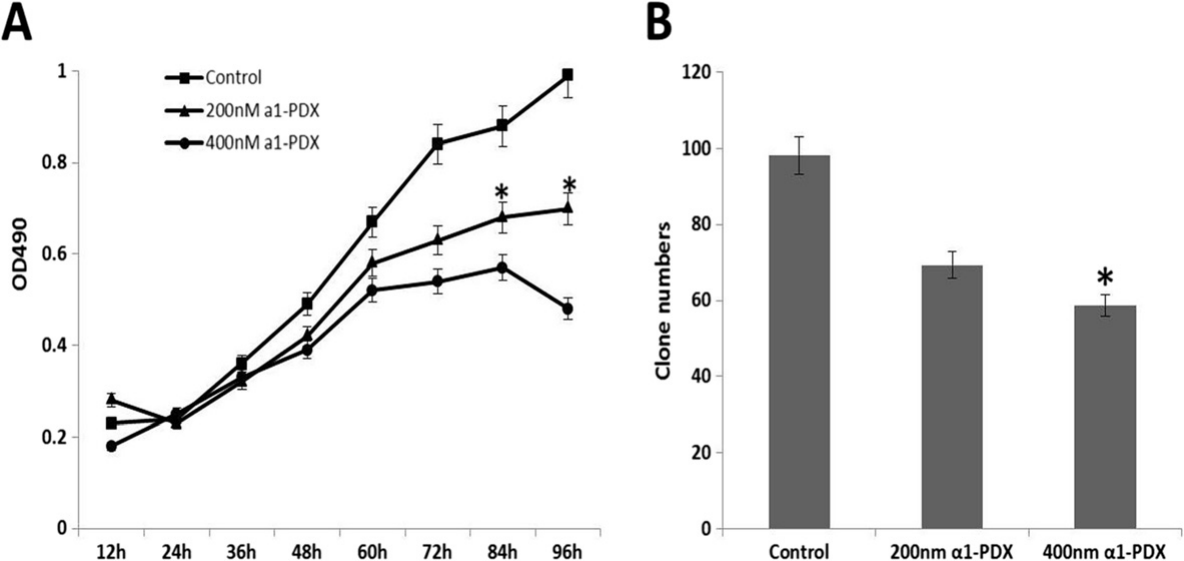
**Effect of a1-PDX on the proliferation of A549 cell.** A549 cells were treated with a1-PDX for indicated times, the proliferation was detected with MTT assay **(A)**. Individual cell was seeded on the 6-well plate, followed with colony analysis **(B)**. Data are expressed as overall Mean ± SE from three independent experiments. (*P < 0.05, vs control).

A549 cell growth was inhibited upon treatment with a1-PDX for over 48 hours (Figure 1A). Then, colony formation assay was performed to detect the clones of A549 cell. The result showed that the colony formation ability of A549 cell was decreased significantly compared to the control group (Figure 1B).

### Effects of a1-PDX on apoptosis of A549 cells

**Figure 2:**
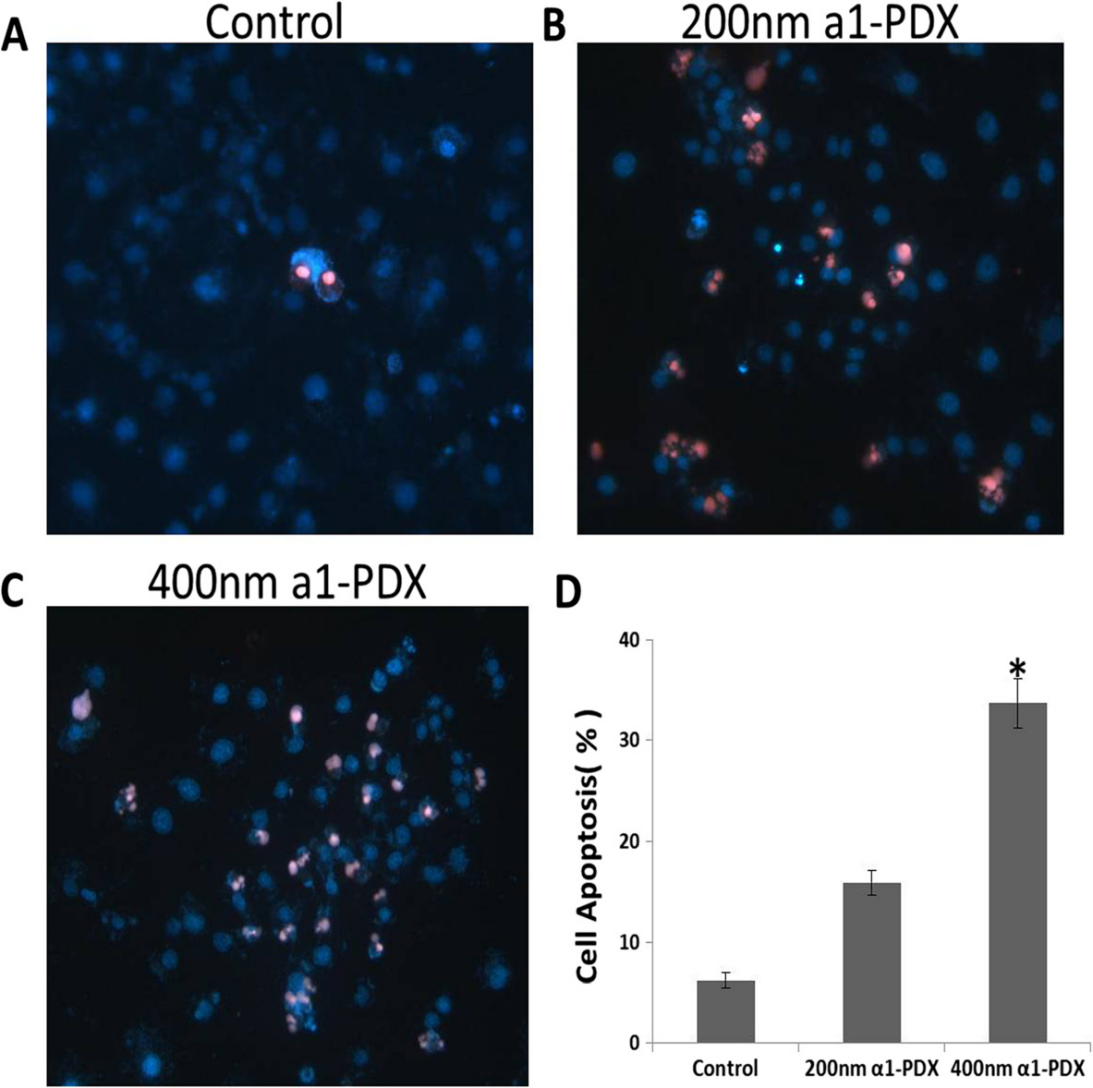
**A1-PDX induce apoptosis in A549 cells.** Cell apoptosis was determined by Hoechst/PI double staining **(A, B, C, D)**. Data are expressed as Mean ± SE from three independent experiments. (*P < 0.05 vs control).

To explore the mechanism of a1-PDX on A549 cell growth inhibition, we first used Hochest33342/PI double staining to detect the morphological change of apoptosis cells. The results showed that a1-PDX can induce apoptosis of A549 cells (Figure 2).

### Effects of a1-PDX on A549 cell migration and invasion

**Figure 3:**
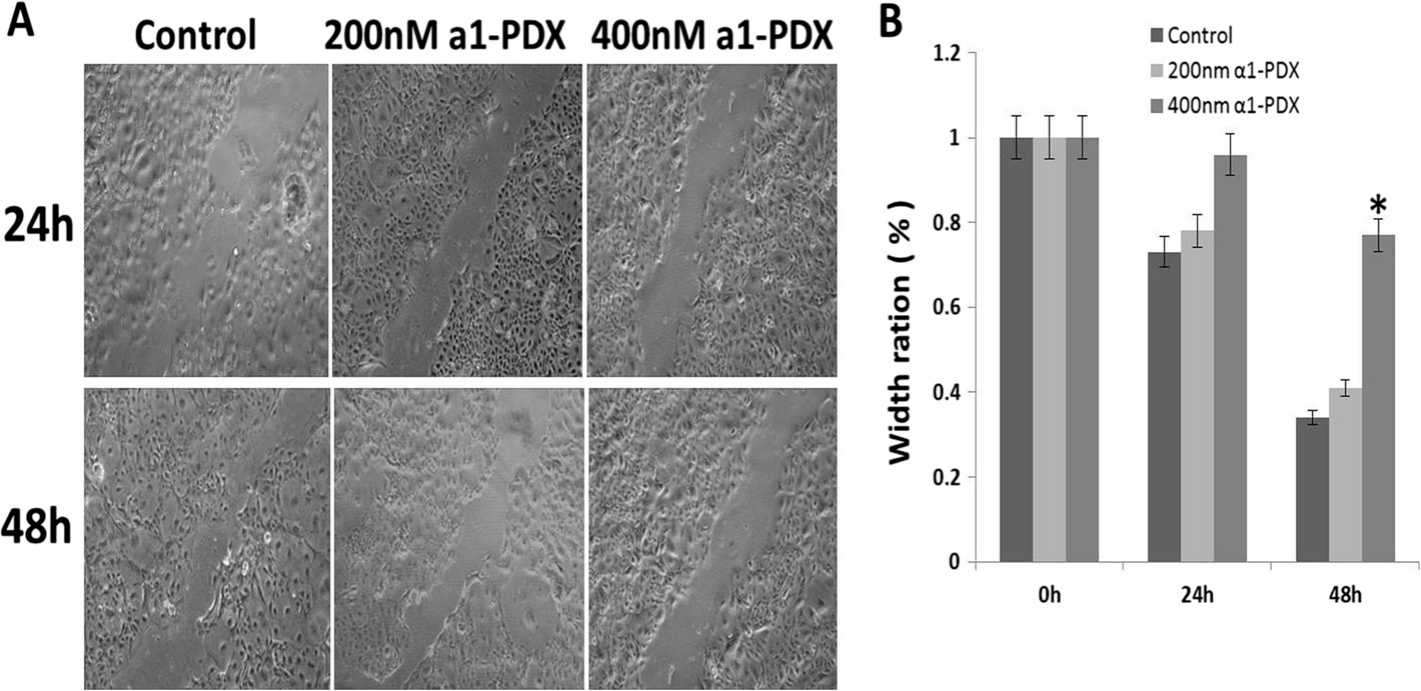
**Effect of a1-PDX on the cell migration by wound healing assay.** Monolayer A549 cell grew to 100% confluent and wounded by a sterile 200 μl pipette tip, the ability of cell migration was monitored with an inverted microscopy equipped with a digital camera **(A)**. Statistical analysis of three independent experiments **(B)** (*P < 0.05 vs control).

**Figure 4:**
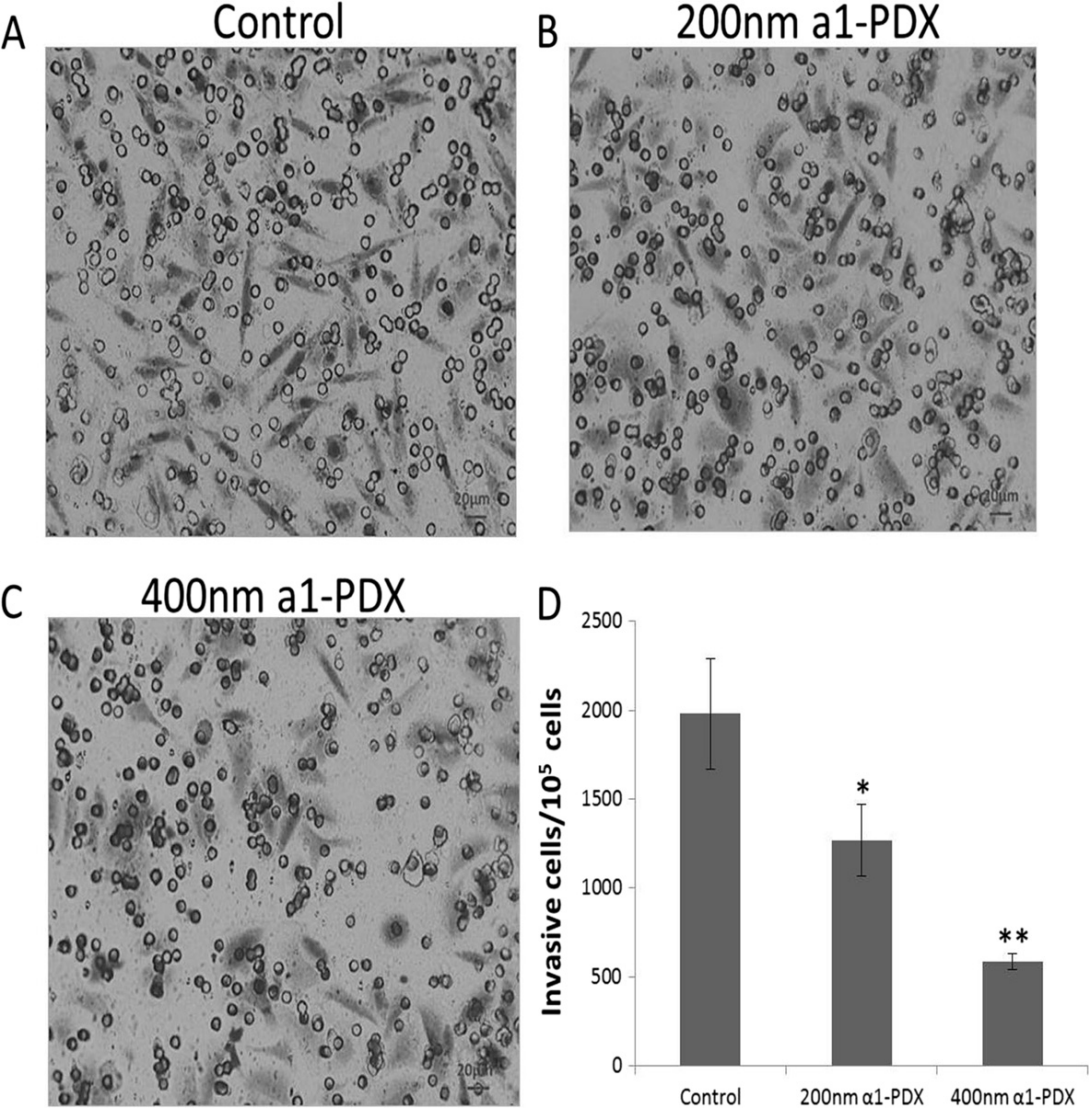
**A549 cell invasion ability upon a1-PDX treatment.** The invasion cells were detected by Transwell assay **(A, B, C)** and counted for statistical analysis **(D)** (*P < 0.05, **P < 0.01 vs control).

To study whether the migration and invasion of A549 cells were regulated by a1-PDX, we then used wound healing and Transwell assays. Two experimental results showed that the migration and invasion capabilities of A549 cells were reduced evidently by a1-PDX compared to the control group (Figures 3 and 4).

**Figure 5:**
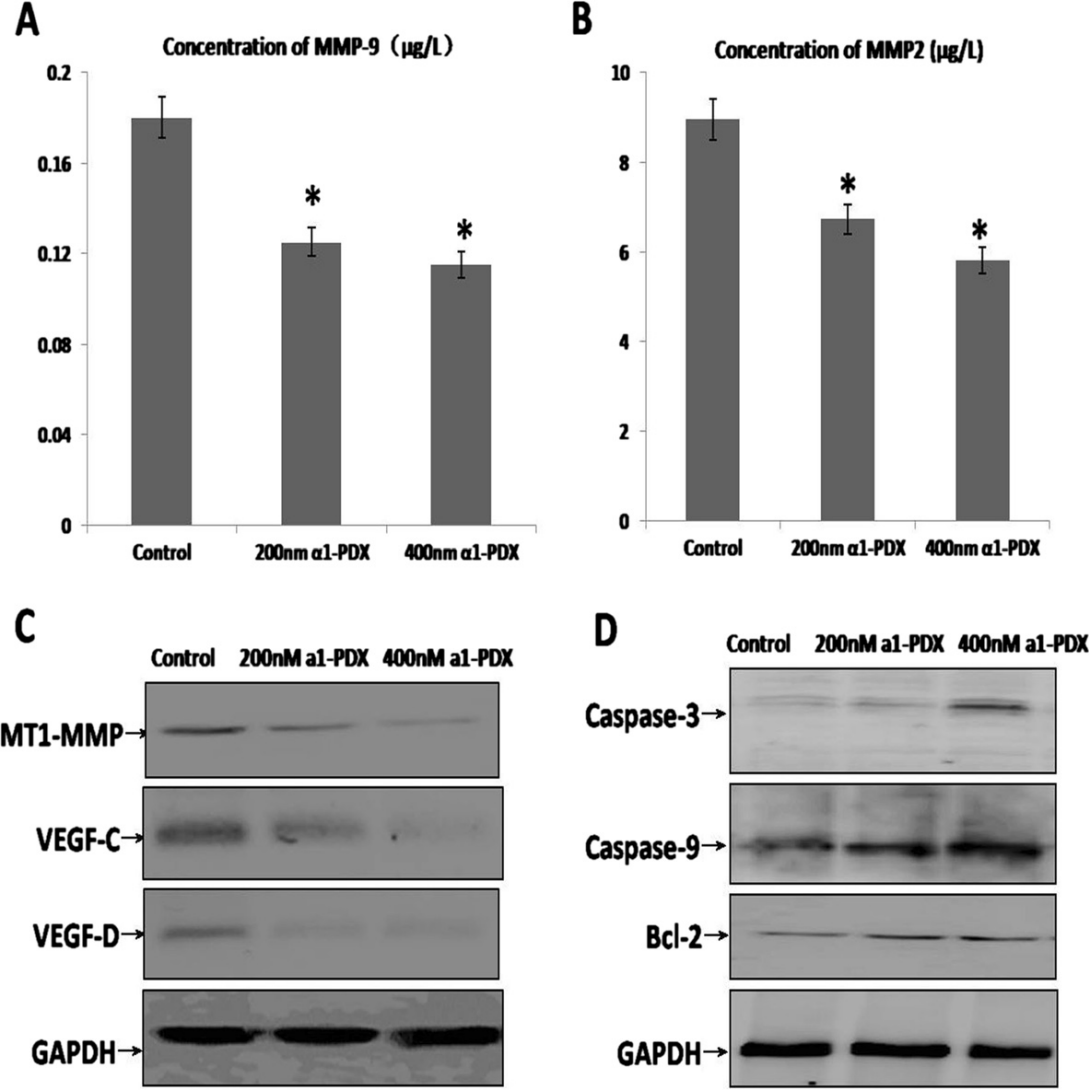
**Effect of a1-PDX on the expression of migration and apoptosis proteins in A549 cells.** MMP2 and MMP9 in SW480 cell culture medium were detected by ELISA assay **(A and B)**. The data represent the Mean ± SE from three independent experiments. (*P < 0.05 vs control ).The expression of cell migration and apoptosis proteins in A549 cells were detected with western blot **(C and D)**.

To fully understand the molecular mechanism of A549 cells migration and apoptosis regulated by a1-PDX, we detected the expression of some proteins with Western Blot and ELISA assay (Figure 5). The concentrations of MMP2, MMP9 in cell culture medium reduced significantly compared to the control group (Figure 5A, B). The expression of MT1-MMP, VEGF-c and VEGF-d decreased significantly compared to the control group (Figure 5C). However, the expression of Caspase-3, Caspase-9 and Bcl-2 were increased evidently in a1-PDX treated groups (Figure 5D).

## Discussion

Tumor metastasis is a complex multi-step process. Many proteins involve in the process of tumor metastasis. As we all know, the tumor extracellular matrix digestion is the most common prerequisite for tumor invasion and metastasis. Tumor cells secrete MMP family members (MMPs) could degrade all of the important extracellular matrix components [11,12]. Thus, the expression of matrix metalloproteinases could effectively reflect the ability of tumor cell invasion. It was proven that matrix metalloproteinase MMP2 and MMP9 degraded the basement membrane was a crucial step that promote tumor invasion and metastasis in many types of tumors [13].

In our current study, a1-PDX could not only inhibit the expression of cell migration proteins, but also increase the expression of cell apoptosis proteins, that induced cell apoptosis. MT1-MMP was Furin substrates, so MT1-MMP precursor was required to be cut by Furin before the maturation [4]. A1-PDX reduced the enzymic activity of Furin, thus reducing the maturation and activation of MT1-MMP. It was further suppressed the maturation of MMP2 and MMP9.

The enhanced expression of VEGF-C and VEGF-D and the lymphatic metastasis of tumor cells had been considered as a prognostic indicator of several types of cancers [14,15]. Their precursors needed to be cut by Furin before maturation and activation [16]. Therefore, we also have examined the expression of VEGF-C and VEGF-D protein in A549 cells which were treated by a1-PDX. The results show that the expressions of VEGF-C and VEGF-D protein are significantly reduced.

## Conclusions

Taken together, we conclude that Furin inhibitor suppressed A549 cell migration through down-regulating the activity of Furin. The subsequent regulation plays a critical role in A1-PDX-induced migration inhibition and apoptosis in A549 cells. Furin inhibitor may, therefore, have a potential use as a target in the treatment of lung cancer.

## Abbreviations

NSCLC: Non-small cell lung cancer; VEGF: Vascular endothelial growth factor; MMPs: Matrix metalloproteinases.

## Competing interests

The authors declare that they have no competing interests.

## Authors’ contributions

YCM, STX designed research; YCM, WJF and SMR performed the experiments and data analysis; LG and ZYB contributed new reagents and analytic tools; YCM, STX wrote the paper. All authors read and approved the final manuscript.
